# Peptide-Directed Synthesis of Aggregation-Induced Emission Enhancement-Active Gold Nanoclusters for Single- and Two-Photon Imaging of Lysosome and Expressed α_v_β_3_ Integrin Receptors

**DOI:** 10.1021/acs.analchem.4c00321

**Published:** 2024-05-23

**Authors:** Manivannan Madhu, Wei-Bin Tseng, Yi-Shiuan Chou, A. Santhana Krishna Kumar, Chi-Yu Lu, Po-Ling Chang, Wei-Lung Tseng

**Affiliations:** † Department of Chemistry, National Sun Yat-sen University, No. 70 Lienhai Rd., Kaohsiung 80424, Taiwan; ‡ Department of Environmental Engineering, Da-Yeh University. No. 168, University Road, Dacun, Changhua 515006, Taiwan; § School of Pharmacy, Kaohsiung Medical University, No. 100, Shiquan first Road, Sanmin District, Kaohsiung 80708, Taiwan; ∥ Faculty of Geology, Geophysics and Environmental Protection, AGH University of Science and Technology, Al. Mickiewicza 30, 30-059 Krakow City, Poland; ⊥ School of Pharmacy, College of Pharmacy, Kaohsiung Medical University, No. 100, Shiquan first Rd., 80708 Kaohsiung, Taiwan

## Abstract

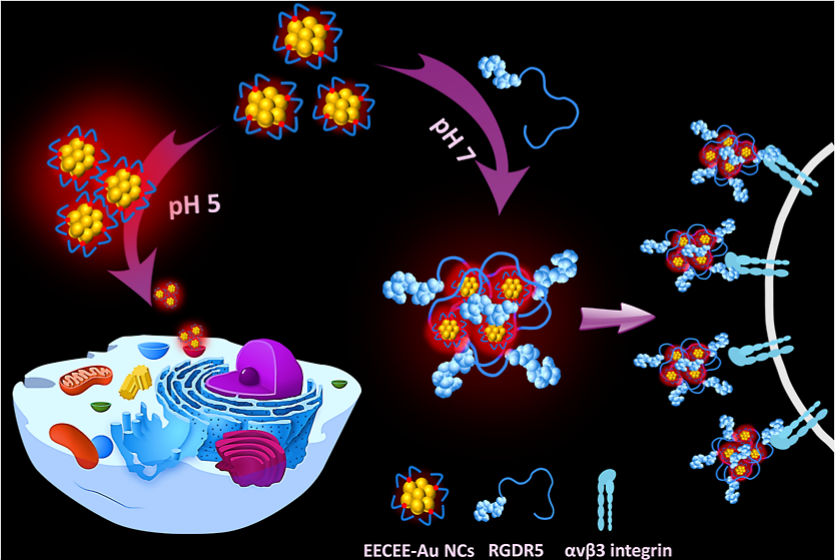

This study explores the synthesis and characterization of aggregation-induced emission enhancement (AIEE)–active gold nanoclusters (AuNCs), focusing on their near-infrared luminescence properties and potential applications in biological imaging. These AIEE-active AuNCs were synthesized via the NaBH_4_-mediated reduction of HAuCl_4_ in the presence of peptides. We systematically investigated the influence of the peptide sequence on the optical features of the AuNCs, highlighting the role of glutamic acid in enhancing their quantum yield (QY). Among the synthesized peptide-stabilized AuNCs, EECEE-stabilized AuNCs exhibited the maximum QY and a pronounced AIEE effect at pH 5.0, making them suitable for the luminescence imaging of intracellular lysosomes. The AIEE characteristic of the EECEE-stabilized AuNCs was demonstrated through examinations using transmission electron microscopy, dynamic light scattering, zeta potential analysis, and single-particle imaging. The formation of the EECEE-stabilized AuNCs was confirmed by size-exclusion chromatography and mass spectrometry. Spectroscopic and electrochemical examinations uncover the formation process of EECEE-stabilized AuNCs, comprising EECEE-mediated reduction, NaBH_4_-induced nucleation, complex aggregation, and subsequent cluster growth. Furthermore, we demonstrated the utility of these AuNCs as luminescent probes for intracellular lysosomal imaging, leveraging their pH-responsive AIEE behavior. Additionally, cyclic arginylglycylaspartic acid (RGD)-modified AIEE dots, derived from cyclic RGD-linked peptide-induced aggregation of EECEE-stabilized AuNCs, were developed for single- and two-photon luminescence imaging of α_v_β_3_ integrin receptor-positive cancer cells.

## Introduction

Tang’s team’s breakthrough discovery of aggregation-induced emission (AIE) in 2001 marked a new era in the development of fluorophores.^1^ The aggregation of AIE-active fluorophores efficiently enhances their emission intensity, transitioning from a nonemissive state to a highly emissive state and causing a shift in the maximum emission wavelength.^2^ This phenomenon stems from AIE-active fluorophores typically incorporating bulky groups, allowing them to maintain a more compact structure to overcome the π–π stacking interactions in the aggregation state. This unique behavior sets AIE-active fluorophores apart from conventional fluorophores, in which π–π stacking interactions quench their fluorescence after aggregation.^2^ Therefore, the difference in fluorescence properties of AIE-activated fluorophores between the dispersed and aggregated states can be exploited to design fluorescence turn-on sensors in response to external stimuli.^3^ Also, the concept of AIE can be extended to ligand-conjugated luminescent nanomaterials, including metal nanoclusters (MNCs),^4^ carbon dots,^5^ polymer dots,^6^ and semiconductor quantum dots.^7^ In contrast to AIE, “aggregation-induced emission enhancement (AIEE)” is preferable to describe the transition from weak to strong emission when ligand-conjugated luminescent nanomaterials are assembled into aggregates. Similarly, the AIEE-based nanosensors can be created by monitoring analyte- and environmental-change-induced luminescence enhancement of ligand-conjugated nanomaterials.

Since molecule-like properties in MNCs^8^ make them stand out from other AIEE-related nanomaterials, research efforts have indeed been dedicated to uncovering and reporting the presence of AIEE properties in MNCs in recent years. These AIEE-active MNCs are made up of a few to several hundred metal atoms, with sizes similar to the electron’s Fermi wavelength (size < 2 nm), and are covered by a monolayer of organic ligands. Their effective electronic transitions between discrete energy levels give them unique photophysical properties, including large Stokes shift, microsecond photoluminescence lifetime, excellent photostability, and significant two-photon absorption (TPA) cross-section.^9^ To induce MNCs by AIEE, precursor metal ions are initially reduced with capping ligands or NaBH_4_ in the presence of capping ligands.^10^ The formed AIEE-active MNCs comprise a metal core encased within a metal(I)-ligand complex layer. After that, the AIEE-active MNCs are assembled by external stimuli such as solvent,^11^ pH,^12^ metal ions,^13^ peptides,^14^ and nanoparticles.^15^ Moreover, the crystallization of MNCs^16^ and their placement into scaffolds (e.g., metal/covalent organic frameworks and zeolites)^17^ is another strategy that can induce AIEE. The assembled MNCs exhibit higher quantum yield (QY), longer PL lifetime, and shorter emission wavelengths than dispersed MNCs. These features allow AIEE-active MNCs to open up a wide range of applications, such as phosphor powders for illumination, in vitro and in vivo imaging, sensor fabrication, and photodynamic antibacterial.^18−20^ However, most AIEE active-MNCs rarely have a maximum emitted wavelength of >700 nm (Table S1), making them susceptible to cellular autofluorescence interference. Although AIEE-active MNCs have been shown to sense pH changes in solution, limited studies have explored the imaging of specific intracellular organelles through pH-induced AIEE of MNCs.^21^ Additionally, the MNCs’ low QYs and low absorption coefficients result in weak brightness, making it impossible to identify them clearly in cellular imaging.

In response to these requirements, AIEE–active gold nanoclusters (AuNCs) were fabricated through the NaBH_4_-mediated reduction of HAuCl_4_ in the presence of peptides. The selection of the AuNCs can be attributable to their aurophilic interaction, which falls within the 5–15 kcal/mol energy range, potentially triggering the AIEE effect. Glutamic acid inclusion in the peptide was crucial for enhancing AuNCs’ QY. In contrast to the other peptide-stabilized AuNCs, EECEE-stabilized AuNCs exhibited superior luminescence regarding the QY and lifetime, mainly showing a significant AIEE effect at pH 5.0. Since the pH of the internal environment of lysosomes ranges from about 4.5 to 5.0,^22^ the EECEE-stabilized AuNCs were well suited for luminescent imaging of intracellular lysosomes. In addition, a peptide with a cyclic RGD moiety and an AIEE active unit was created to activate the AIEE of the EECEE-stabilized AuNCs. The produced cyclic RGD-modified AIEE dots were readily used to image α_v_β_3_ integrin receptor-positive cancer cells through single- and two-photon luminescence detection modes.

## Results and Discussion

### Influence of Peptide Sequence on Optical Properties of the Peptide-Stabilized AuNCs

**Figure 1:**
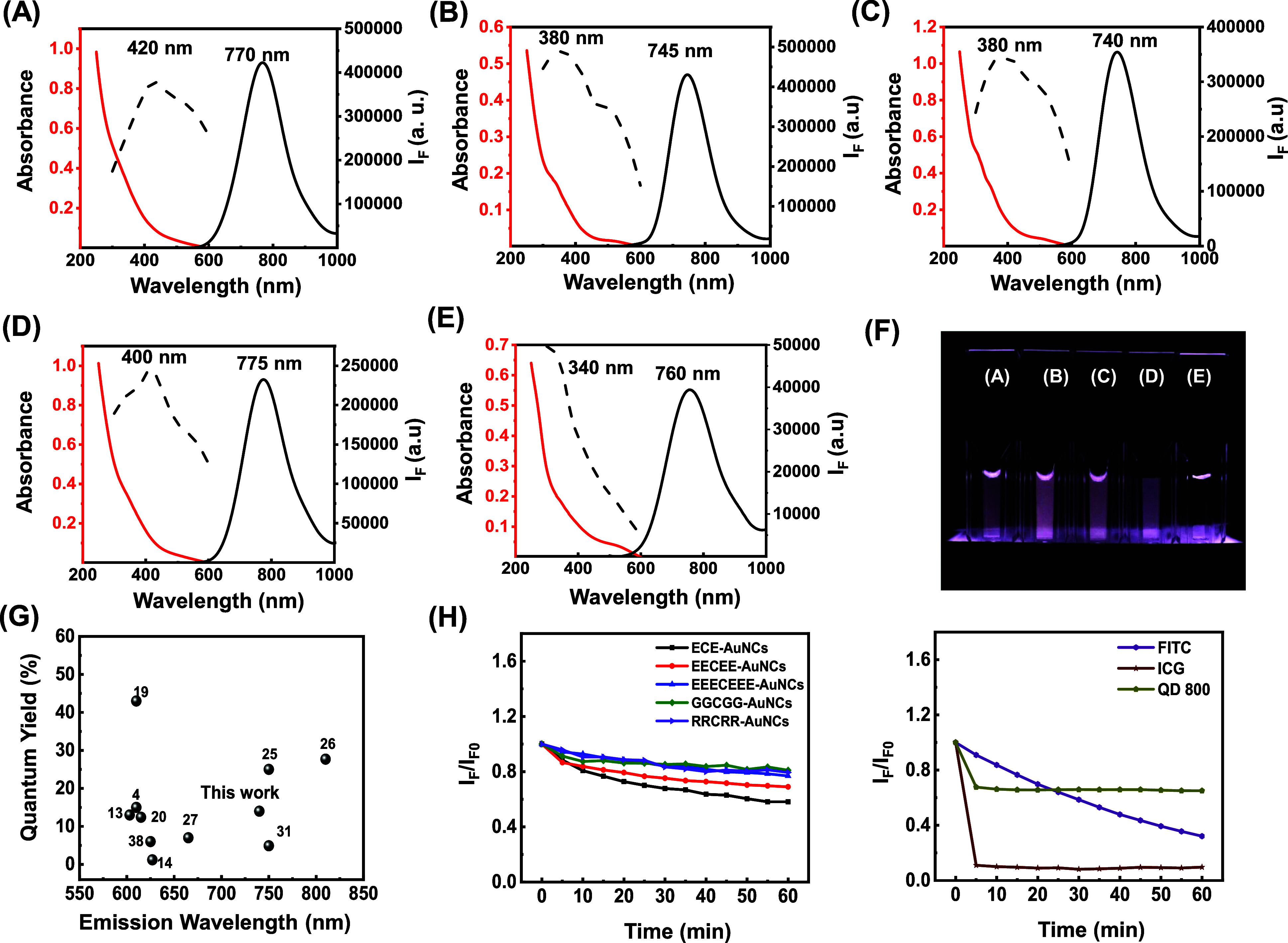
Comparison of optical properties of peptide-stabilized AuNCs. (A–E) Excitation, luminescence, and absorption spectra of (A) ECE-, (B) EECEE-, (C) EEECEEE-, (D) GGCGG-, and (E) RRCRR-stabilized AuNCs. (F) Photographs of the ECE-, EECEE-, EEECEEE-, GGCGG, and RRCRR-stabilized peptides. (G) Comparison of the EECEE-stabilized AuNCs with previously published visible and NIR-emitting AuNCs (collected from refs (4, 13, 14, 19, 20, 25–27, 31 and 38)) regarding maximum emission wavelengths and QYs. (H) Photostability of (left panel) peptide-stabilized AuNCs and (right panel) Qdots800, indocyanine green, and fluorescein isothiocyanate under exposure of 488 nm at ambient temperature.

A previous study reported that ligands with electron-rich groups (e.g., COOH, CONH_2_) or atoms (e.g., N, O) significantly enhance the QY of Au_25_(SR)_18_ through the donation of delocalized electrons from the capping ligand to the gold core.^23^ According to this concept, five different peptides were designed as templates to produce quantum-sized AuNCs, including ECE (pI = 3.80), EECEE (pI = 3.58), EEECEEE (pI = 3.46), GGCGG (pI = 5.52), and RRCRR (pI = 12.0), whose structures are shown in Table S2. The designed peptide facilitates our understanding of the connection between the QY of AuNCs and the side functional groups of the peptide (e.g., COOH). Cysteine (C) in the peptide sequence facilitates binding to AuNC surfaces via a covalent Au–sulfur connection, aiding in both the reduction and nucleation of AuNCs. Glutamic acid (E) and arginine (R) provide negative and positive charges, respectively, enhancing repulsion between AuNCs and stabilizing them in solution. We synthesized NIR-emitting AuNCs by mixing HAuCl_4_, designed peptides, and TCEP solution at 70 °C for 15 min, followed by NaBH4 addition and an 8 h reaction. The resulting ECE-, EECEE-, EEECEEE-, GGCGG-, and RRCRR-stabilized AuNCs exhibited maximum emission wavelengths beyond 740 nm (Figure 1A–E), with EECEE-stabilized AuNCs showing the brightest emission. The excitation spectrum of each AuNC indicated their optimal excitation wavelength (dashed line in Figure 1A–E). Unlike gold nanoparticles with strong surface plasmon resonance in the visible region, the synthesized AuNCs displayed a broad absorption band from the UV to the visible region. Under UV illumination, a camera with a 700–1100 nm filter was used to capture photographs of the identical concentration of the five peptide-stabilized AuNCs (Figure 1F). The photographs show that the EECEE-stabilized Au NCs produced the highest brightness compared to that of the other peptide-stabilized AuNCs. The QYs of the ECE-, EECEE-, EEECEEE-, GGCGG, and RRCRR-stabilized peptides were measured as 7.0, 14, 7.0, 5.6, and 1.8%, respectively, with excitation at 480 nm (Table S3). Evidently, the presence of glutamic acid in the designed peptide significantly enhances the QY of the AuNCs. This enhancement can be attributed to the carboxylic acid group in glutamic acid donating delocalized electrons to the Au core, thereby promoting repulsion between formed AuNCs in an aqueous solution.^24^ The higher number of glutamic acid residues in EECEE compared to ECE likely contributes to its superior QY. However, although EEECEEE contains more glutamic acid residues, the ligand is too bulky. As a result, the number of EEECEEE molecules on the surface of the AuNCs is less than that of EECEE, leading to a lower QY despite the increased glutamic acid content. The QY of the EECEE-stabilized AuNCs was comparable to those of previously published visible and NIR I-emitting AuNCs (Figure 1G).^25,26^

The luminescence decay of peptide-stabilized AuNCs revealed a long average luminescence lifetime (0.6–1.4 μs; Table S3), attributed to ligand–metal charge transfer (LMCT) relaxation of surface Au(I)–S triplet states^27^ or rapid geometrical and electrical changes during photoexcitation.^28^ Under continuous exposure to 488 nm light for 1 h, the photobleaching resistance of peptide-stabilized AuNCs was similar to that of good photobleaching-resistance Qdots800 (CdSe/ZnS core–shell quantum dots)^29^ and superior to that of indocyanine green and fluorescein isothiocyanate (Figure 1H). Due to their superior QY, we opted to use the EECEE-stabilized AuNCs as an imaging reagent.

### Potential Structure of the EECEE-Stabilized AuNCs and Their Formation Mechanism

**Figure 2:**
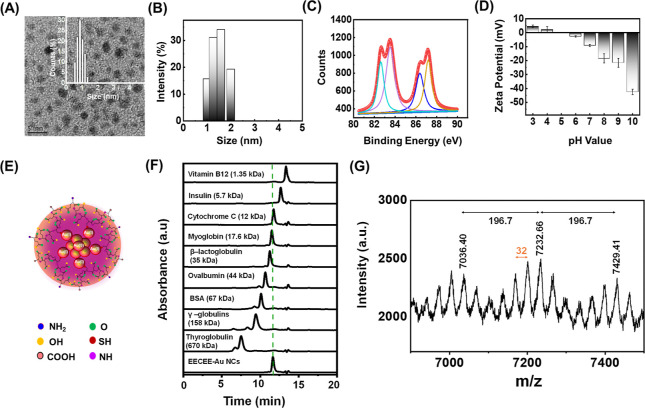
Characterization and structural analysis of the EECEE-stabilized AuNCs. (A) TEM image, (B) DLS spectrum, (C) XPS spectrum, and (D) pH-dependent zeta potentials of the AuNCs. (E) Schematic illustration of the EECEE peptide arrangement on the surface of the AuNCs. (F) SEC chromatograms of the AuNCs and the other standard proteins. (G) MALDI-TOF-MS spectrum of the AuNCs.

The morphology and chemical composition of the EECEE-stabilized AuNCs were verified by transmission electron microscopy (TEM), dynamic light scattering (DLS), X-ray photoelectron spectroscopy (XPS), zeta potential analysis, size-exclusion chromatography (SEC), and matrix-assisted laser desorption/ionization time-of-flight mass spectrometry (MALDI-TOF-MS). TEM and DLS analyses of the EECEE-stabilized AuNCs revealed a core size of 0.9 ± 0.2 nm (Figure 2A) and a hydrodynamic diameter of 1.1 ± 0.2 nm (Figure 2B), respectively, possibly influenced by the capping peptide. The distance between adjacent well-defined crystal planes in the high-resolution TEM images of the proposed AuNCs was determined to be 0.23 nm (inset in Figure 2A), which is characteristic of the Au lattice spacing. As indicated in the XPS spectrum (Figure 2C), the EECEE-stabilized AuNCs exhibited the binding energy of the Au 4f_7/2_ band between 84.0 eV (bulk Au) and 85.0 eV for Au(I)-thiolate complexes,^30^ demonstrating that they comprise both Au(0) core and Au(I) shell. The zero zeta potential of the AuNCs was approximately 5.0 after measurement of their zeta potential as a function of solution pH (Figure 2D). The difference between the zero zeta potential value (5.0) of the AuNCs and the pI (3.58) of the EECEE peptide suggests that the specific arrangement of the EECEE peptide on the surface of the AuNCs leads to exposure of the terminal amino and carboxyl groups to the aqueous environment (Figure 2E). In other words, the zeta potential of EECEE-stabilized AuNCs is determined by their exposed terminal amino and carboxyl groups, not by their peptide sequence. As shown in SEC chromatograms (Figure 2F), the peak of AuNCs was located between cytochrome C (12 kDa) and myoglobin (17.6 kDa), demonstrating the formation of the AuNCs consisting of an inner Au core and several outer Au(I)-EECEE complexes.^24^ MALDI-TOF-MS also characterized the EECEE-stabilized AuNCs with α-cyano-4-hydroxycinnamic acid as the matrix, chosen for its ability to ionize peptide- and protein-stabilized AuNCs.^31^ The mass spectra of the EECEE-stabilized AuNCs revealed multiple peaks with *m*/*z* intervals of 196.9 and 32.0, which correspond to the loss of [Au] and [S], respectively (Figure 2G). The most intense peak, observed at *m*/*z* 7232.67, was identified as [Au_25_S_11_ + 3EECEE – 3H + 2Na]^+^ (molecular weight of EECEE is 637.6). Notably, the laser source’s high energy can induce the AuNCs’ rupture, releasing the EECEE peptide through the cleavage of the peptide’s S–C bond.^32^ Although the MALDI-TOF-MS analysis provided valuable insights into the composition of the AuNCs, it does not definitively confirm the exact configuration or number of atoms within the cluster’s core. Determining the kernel number of Au_25_ nanoclusters requires additional characterization techniques.

The following study investigates the formation mechanism of the EECEE-stabilized AuNCs. A solution of HAuCl_4_ showed an absorption peak at 290 nm (red line Figure S1A), disappearing upon the addition of TCEP-treated EECEE due to Au(III) reduction by the thiol groups of EECEE (green line in Figure S1A). Raman and Fourier transform infrared spectra confirmed the Au–S bond formation at 320 cm^–1^ and the disappearance of the thiol peak around 2550–2600 cm^–1^ in a mixture of TECP-treated EECEE and HAuCl_4_, respectively (Figure S1B,C). The cyclic voltammetry analysis of HAuCl_4_ alone revealed a reduction wave at +0.21 V, while no such wave was observed in the presence of EECEE (Figure S1D). These results indicate that the EECEE peptide not only binds to HAuCl_4_ through the formation of Au–S bonds but also triggers the reduction of Au(III) to Au(I), forming the Au(I)–EECEE complex. After incubating NaBH_4_ with a mixture of HAuCl_4_ and EECEE for 5 min, a broad peak at 335 nm appeared in the absorption spectrum (black line in Figure S1A), resembling those of known Au_10_(glutathione)_10_ and Au_10_(thioglycolic acid)_10._^33,34^ This finding suggests NaBH_4_’s role in promoting Au(I)–EECEE complex formation, which aggregates through Au(I)–Au(I) interactions, initially showing weak luminescence (green line, Figure S1F). Over 1 h (blue line, Figure S1E), absorption characteristics evolved significantly compared to the initial 5 min reaction, with detectable luminescence indicating cluster growth and stabilization. This process continued over 1 to 9 h, as observed in Figure S1E,F. In summary, EECEE-stabilized AuNC formation involves EECEE-mediated reduction, NaBH_4_-triggered nucleation, complex aggregation, and cluster growth, as depicted in Figure S2.

### Luminescence Imaging of Intracellular pH and Lysosomes

**Figure 3:**
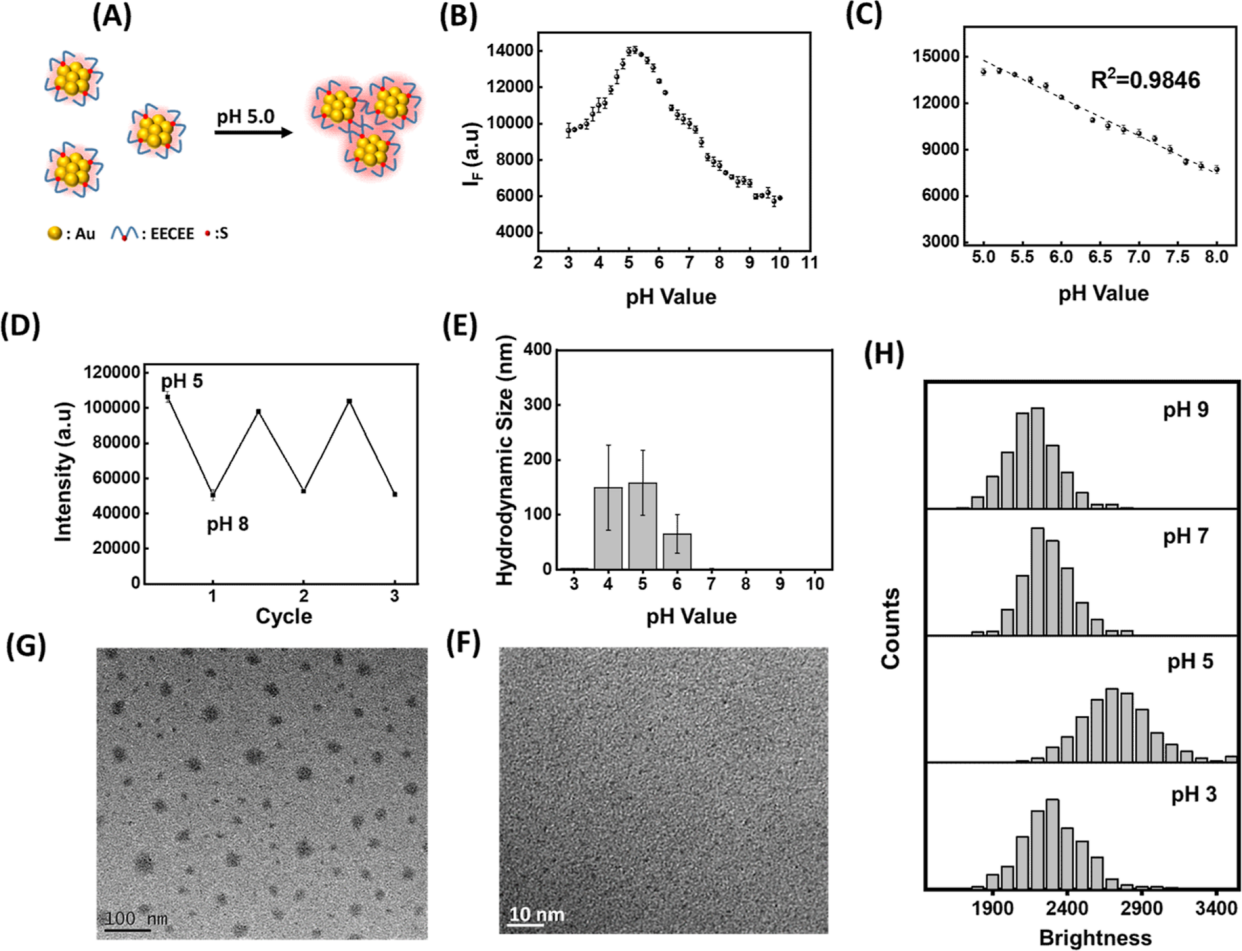
Characterization of the pH-responsive luminescent probe properties of the EECEE-stabilized AuNCs. (A) pH-induced AIEE of AuNCs at pH 5.0. (B) Luminescence intensity (745 nm) of the AuNCs across pH 3.0 to 10.0 in the 25 mM phosphate buffer. (C) A plot of the solution pH versus the AuNCs’ luminescence intensity at 745 nm. (D) AuNCs’ luminescence intensity measured at 745 nm while cyclically adjusting solution pH between 5.0 and 8.0. (E) Hydrodynamic diameters of the AuNCs at different pH. (F,G) TEM images of the AuNCs at pH 5.0 and 8.0. (H) Brightness distribution of 500 individual AuNCs versus pH.

The lysosomal pH typically falls around 5.0, and lysosomal dysfunction can lead to various diseases.^22^ Given the EECEE-stabilized AuNCs’ zero zeta potential around 5.0 (Figure 2D), they may self-assemble into bright aggregates in lysosomes (Figure 3A). This pH-active AIEE effect arises from the restriction of intramolecular motion of the capping ligands around the Au core upon aggregation.^14^ The luminescence intensity test in a pH range of 3.0 to 10.0 revealed maximum brightness at pH 5.0 (Figure 3B), suggesting the potential of the EECEE-stabilized AuNCs for lysosomal imaging; their corresponding luminescence spectra are shown in Figure S3. The luminescence intensity of the EECEE-stabilized AuNCs correlated linearly with pH from 5.0 to 8.0 (*R*^2^ = 0.9846), with an interval of 0.2 pH units and excellent repeatability (relative standard deviation < 5%). Although other reported pH-sensitive AuNC probes showed a similar range of pH variation, their maximum emission wavelength is below 700 nm (Table S4). Figure 3D displays that the luminescence intensity of the EECEE-stabilized AuNCs was fully reversible in successive pH cycles between pH 5.0 and 8.0, indicating reversible self-assembly with pH changes. The minor fluctuation in luminescence intensity could be attributed to the slight increase in the AuNC volume after adding NaOH and HCl. The luminescence intensity peak of the EECEE-stabilized AuNCs at pH 5.0 was investigated using DLS to estimate their hydrodynamic diameter at different pH values. Figure 3E shows a significant increase in diameter at pH 4.0, 5.0, and 6.0, with the most pronounced increase observed at pH 5.0, indicating that the EECC-stabilized AuNCs aggregated to the highest extent at pH 5.0. Electrostatic repulsion between the AuNCs at pH 3.0 and 7.0 prevented aggregation due to their surface positive and negative charges, respectively. However, at pH 4.0, 5.0, and 6.0, insufficient repulsion led to severe aggregation, corroborating the luminescence intensity peak of the AuNCs at pH 5.0, attributed to AIEE. TEM images of the EECEE-stabilized AuNCs supported DLS findings, showing their aggregation at pH 5.0 (Figure 3F) and dispersion at pH 8.0 (Figure 3G). Single-particle imaging allows us to examine the AIEE behavior of individual AuNCs without interference from neighboring particles or bulk effects, which is essential for understanding the underlying mechanisms of the aggregation and dispersion of AuNCs at different pH values. Videos S1–S7 captured time-evolving luminescence images of individual AuNCs in the pH range of 3.0 to 9.0 using an excitation wavelength of 488 nm and an emission range of 700–800 nm. Analysis of 500 individual AuNCs revealed the highest brightness distribution at pH 5.0 (Figure 3H), indicating that the AIEE behavior of AuNCs is pH dependent. This finding aligns with earlier observations of the luminescence intensity responsiveness to pH changes in bulk solution.

Previous studies indicate that intracellular glutathione and adenosine triphosphate concentrations range from 1 to 10 mM,^35^ potentially affecting the ability of EECEE-stabilized AuNCs to sense intracellular pH. However, Figure S4 demonstrates that even in the presence of 5 mM glutathione and 5 mM adenosine triphosphate, the AuNCs maintain an excellent linear correlation between luminescence intensity and solution pH, suggesting retention of pH sensitivity in live cells. A 3-(4,5-dimethylthiazol-2-yl)-2,5-diphenyltetrazolium bromide (MTT) assay on HeLa cells exposed to various AuNC concentrations (65, 33, and 16 μg/mL) for 6, 12, and 24 h (Figure S5) revealed cell viability consistently above 85%, indicating excellent biocompatibility. To assess the effect of intracellular pH changes on the AuNC luminescence, HeLa cells were treated with the EECEE-stabilized AuNCs for 6 h, washed using phosphate-buffered solution (PBS) at different pH values (5.0–7.5), and then treated with formaldehyde solution at the same pH to induce intracellular pH changes. Confocal laser scanning microscopy (CLSM) images (Figure S6A) showed a decrease in luminescence intensity as pH varied from 5.0 to 7.5, consistent with low pH-induced AIEE of the AuNCs. Plotting the average luminescence intensity of 100 HeLa cells versus intracellular pH revealed a good linear relationship (Figure S6B), demonstrating AuNCs’ ability to probe intracellular pH changes by monitoring luminescence intensity. However, changing intracellular pH with PBS may disrupt organelles, potentially leading to cell death due to inconsistencies with the intracellular environment.

**Figure 4:**
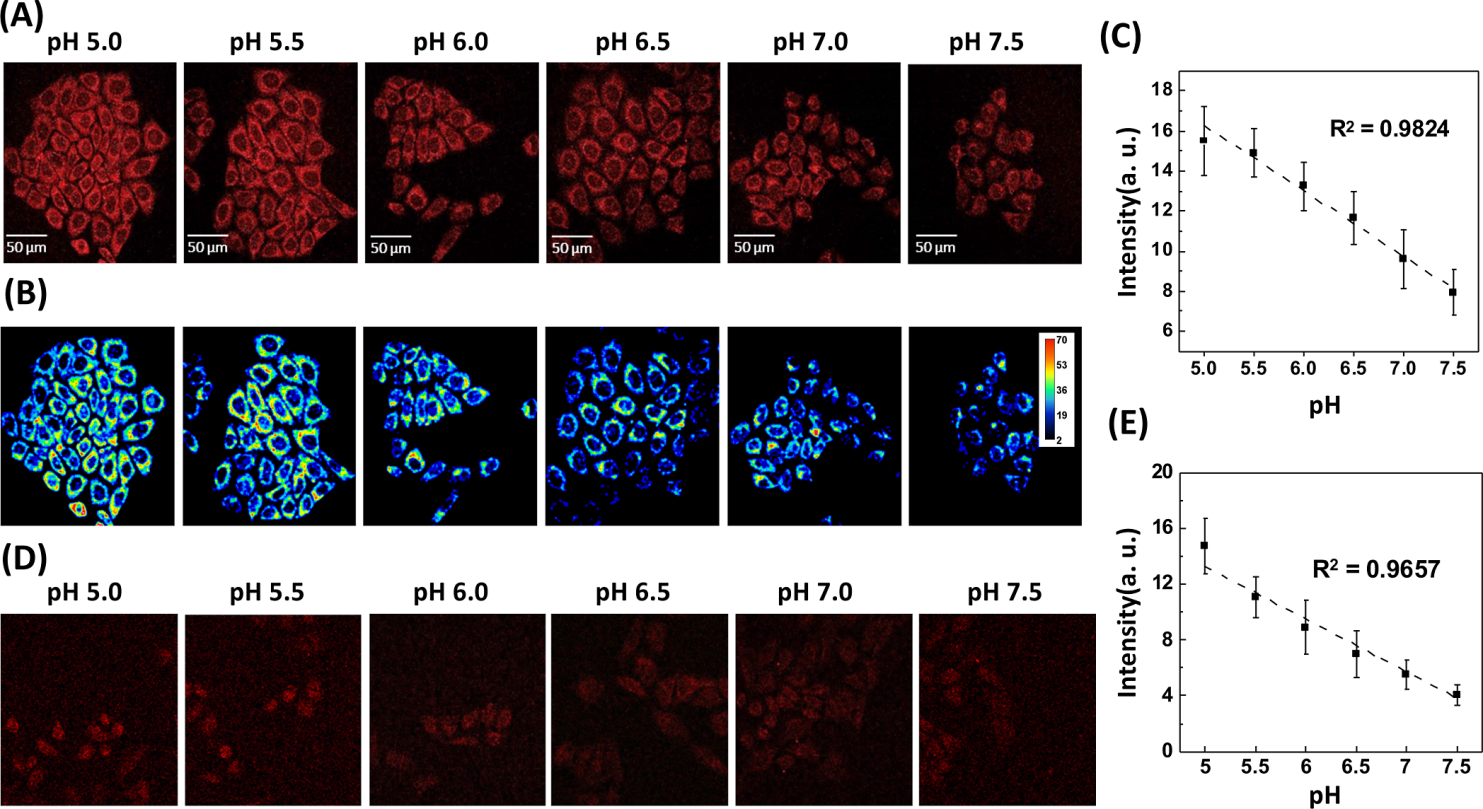
Luminescence imaging of intracellular pH changes with the EECEE-stabilized AuNCs while incubating HeLa cells with pH calibration curve buffers. (A) CLSM, (B) pseudocolor, and (D) two-photon images of HeLa cells cultured with different pH calibration curve buffers followed by labeling with the EECEE-stabilized AuNCs. (C,E) Mean intracellular luminescence intensity obtained from the (C) CLSM and (E) two-photon images of the labeled HeLa cells (*n* = 100) at different pH values. The excitation wavelength was 488 and 840 nm for single- and two-photon images, respectively. The luminescence of the EECEE-stabilized AuNCs was collected in the NIR channel (700–800 nm).

A pH calibration curve buffer containing monensin was prepared to regulate intracellular pH without disrupting cellular function, facilitating cation transport across cell membranes in an electroneutral environment and maintaining intracellular/extracellular pH balance.^36^ The following steps of washing and fixing the cells are the same as the previous steps of changing the pH of the cells with a PBS solution. The CLSM images (Figure 4A) of EECEE-stabilized AuNC-labeled cells and corresponding pseudocolor representations (Figure 4B) demonstrated a progressive reduction in luminescence intensity of HeLa cells as pH decreased from 5.0 to 7.5. Analysis of the red luminescence intensity of 100 HeLa cells at varying pH values showed a good linear relationship with intracellular pH (Figure 4C), enabling the tracking of acidic organelles in future experiments. Considering the remarkable TPA cross-section properties of glutathione-stabilized AuNCs,^37^ it is reasonable to assume similar properties for the EECEE-stabilized AuNCs. Two-photon excitation imaging, offering advantages over single-photon excitation,^9^ such as reduced tissue/cell scattering and autofluorescence, was conducted after regulating intracellular pH with the pH calibration curve buffer. Two-photon luminescence images (Figure 4D) revealed a gradual decrease in the luminescence intensity of EECEE-stabilized AuNCs within cells as the pH increased from 5.0 to 7.5. Calculations of luminescence intensity from 50 cells demonstrated a linear correlation with intracellular pH (Figure 4E), indicating a sufficient TPA cross-section of the EECEE-stabilized AuNCs for detectable two-photon luminescence signal generation upon excitation at 840 nm. Our observations revealed the highest luminescence intensity of EECEE-stabilized AuNCs at an intracellular pH of 5.0, suggesting potential accumulation and luminescence enhancement upon entering lysosomes. To confirm this, cells were coincubated with Lysotracker Green (a lysosome-selective dye)^38^ and EECEE-stabilized AuNCs. The CLSM images demonstrated a high degree of overlap between Lysotracker Green fluorescence and AuNC luminescence after just 30 min of incubation (Figure S7), indicating the rapid internalization of AuNCs by lysosomes. Lysosomal colocalization analysis was verified after 6 h of endocytosis. The pixel intensities of the line profiles on the green and red emission images are analyzed by setting straight lines a and c in Figure S8A and lines b and d in Figure S8B. The merge images displaying the overlap between Lysotracker green- and EECEE-stabilized AuNCs-labeled cells are shown in Figure S8C. Although the intensity of Lysotracker green was lower than that of the AuNCs, most of the green signal of Lysotracker green overlapped with the red one of the EECEE-stabilized AuNCs (Figure S8D,E). Further analysis of the colocalization using ImageJ showed a Pearson’s correlation coefficient of 0.813 for the overlap of green and red light (Figure S8F), confirming that the proposed AIEE-active AuNCs indeed accumulate in lysosomes.

### Targeting Imaging of α_v_β_3_ Integrin Receptors-Overexpressed Cells

**Figure 5:**
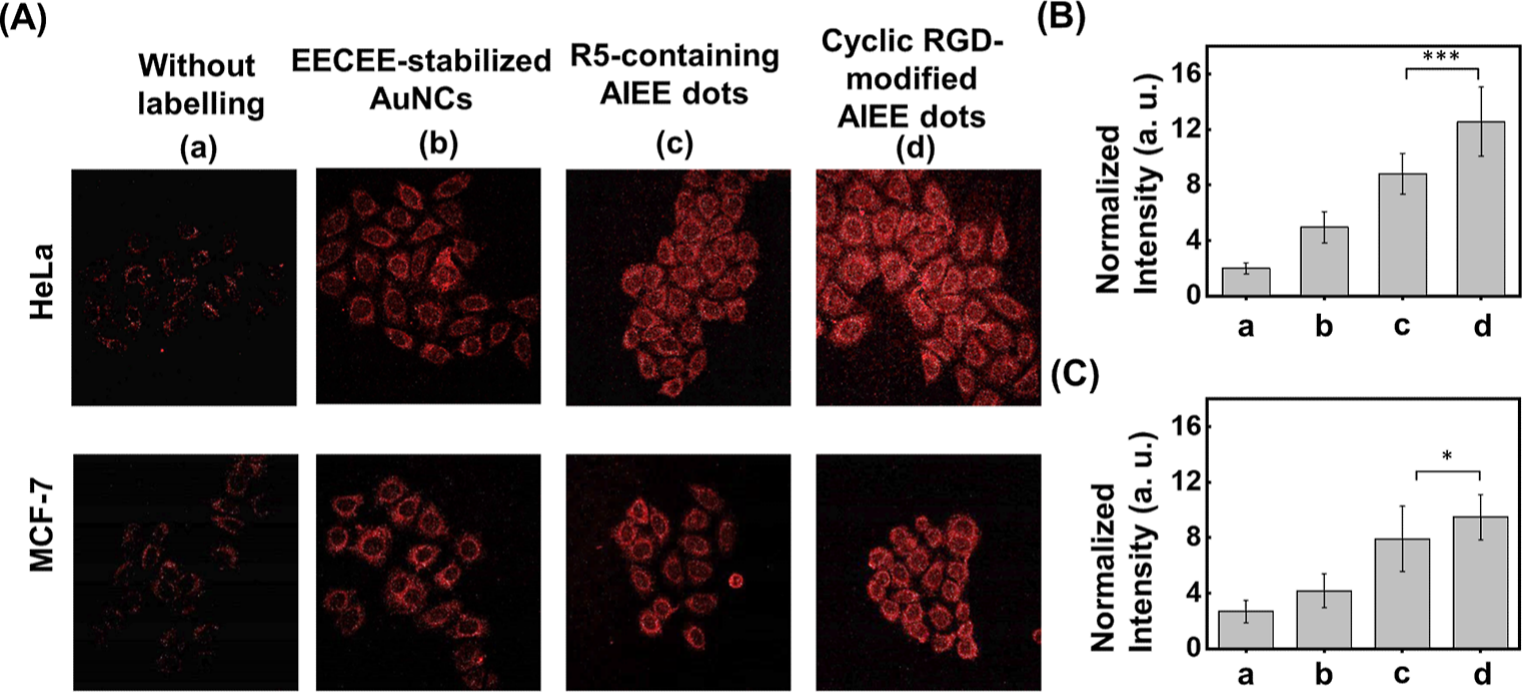
Comparative analysis of intracellular labeling efficacy using EECEE-stabilized AuNCs, R5-containing AIEE dots, and cyclic RGD-modified AIEE dots in the three cancer cells. (A) CLSM images of HeLa and MCF-7 cells labeled (a) without and (b–d) with (b) the EECEE-stabilized AuNCs, (c) the R5-containing AIEE dots, and (d) the cyclic RGD-modified AIEE dots. (B,C) Mean intracellular normalized fluorescence intensity of (B) HeLa and (C) MCF-7 cells (*n* = 100) labeled (a) without and (b–d) with (b) the EECEE-stabilized AuNCs, (c) the R5-containing AIEE dots, and (d) the cyclic RGD-modified AIEE dots. The autofluorescence intensity of the cells without labeling was set as a reference point with a value of 1.0, and the fluorescence intensity of the other labeled cells was measured relative to this reference. The excitation wavelength was set to 488 nm. The fluorescence was collected in the NIR channel (700–800 nm). The symbols *** and * denote *p*-values less than 0.001 and 0.1, respectively.

The EECEE-stabilized AuNCs, exhibiting enhanced luminescence through low pH-induced aggregation, interact with cyclic RGD-modified five-repeat arginine peptides (RGDR5) at neutral pH to form AIEE dots, potentially targeting α_v_β_3_ integrin-overexpressed cells (Figure S9A).^14,39^ It is noted that α_v_β_3_ integrin receptor is frequently identified to be overexpressed in various tumor cells while being less expressed in normal cells.^40^ Optimization experiments determined the optimal concentration (35 μM) of the cyclic RGDR5 peptide to induce maximal luminescence enhancement of the AuNCs (Figure S10). The resulting cyclic RGD-modified AIEE dots displayed approximately 2.2-fold enhanced luminescence, with a blue shift in emission peak, improved QY, and extended luminescence lifetime compared with EECEE-stabilized AuNCs (Figure S9B and Table S3). The AIEE dots had a mean size of 192 ± 35 nm (*n* = 50; Figure S9C), a hydrodynamic diameter of 256 ± 74 nm (Figure S9E), and a zeta potential of +5.7 mV at pH 7.0. The high-resolution TEM images demonstrate that the AIEE dots consist of numerous smaller AuNCs (Figure S9D). A control experiment was conducted using positively charged R5 without RGD to induce aggregation of the EECEE-stabilized AuNCs. The formed R5-containing AIEE dots were then characterized by electron microscopy and spectroscopy-related methods (Figure S11), with details of morphology and optical properties summarized in Table S3. The cyclic RGD-modified and R5-containing AIEE dots exhibited good biocompatibility (>85% cell viability after 24 h) (Figure S12). Afterward, the same concentrations of the EECEE-stabilized AuNCs, R5-containing AIEE dots, and cyclic RGD-modified AIEE dots were incubated separately with HeLa and MCF7 cells for 6 h. The difference between α_v_β_3_ integrin-positive HeLa cells and α_v_β_3_ integrin-negative MCF-7 cells allows us to examine the selectivity of the cyclic RGD-modified AIEE dots toward α_v_β_3_ integrin-expressed cancer cells.^41,42^ The CLSM images obtained in HeLa cells show that the cyclic RGD-modified AIEE dots displayed the highest luminescence intensity, followed by the R5-containing AIEE dots and EECEE-stabilized AuNCs (Figure 5A). Compared with negatively charged EECEE-stabilized AuNCs, the positive charge on the surface of the cyclic RGD-modified and R5-containing AIEE dots can interact favorably with negatively charged cell membranes, facilitating their uptake and increasing their luminescence intensity.^43^ Furthermore, the cyclic RGD-modified AIEE dots can selectively target HeLa cells more effectively than R5-containing AIEE dots due to the expression of the α_v_β_3_ integrin in HeLa cells. A *t*-test confirmed a significant difference in intracellular luminescence intensity between cyclic RGD-modified and R5-containing AIEE dots (Figure 5B). By contrast, no significant difference between cyclic RGD-modified and R5-containing AIEE dots was observed in MCF-7 cells according to the CLSM images (Figure 5A) and a *t*-test (Figure 5C). Due to no expression of α_v_β_3_ integrin receptors on the surface of MCF7 cells, the cyclic RGD-modified AIEE dots are unlikely to enter the cells via receptor-mediated endocytosis. Therefore, both AIEE dots may be internalized through nonspecific endocytic pathways, such as clathrin-mediated or caveolae-mediated endocytosis. Two-photon microscopy further validated the preferential uptake of cyclic RGD-modified AIEE dots by HeLa cells over MCF7 cells (Figure S13), highlighting their potential for targeted imaging in α_v_β_3_ integrin-positive cancer cells.

## Conclusions

This study has demonstrated the EECEE-mediated synthesis of AIEE-active AuNCs with a maximum emission wavelength of 740 nm and suggested their possible formation mechanism and geometry. The EECEE-stabilized AuNCs exhibit the highest luminescence intensity at pH 5.0 due to their aggregation caused by the zeta potential approaching zero, leading to their self-assembly into bright aggregates in lysosomal organelles through the pH-active AIEE effect. Moreover, the incorporation of cyclic RGD with the EECEE-stabilized AuNCs generated high-brightness cyclic RGD-modified AIEE dots, which were shown to be powerful for single- and two-photon luminescence imaging of highly expressed α_v_β_3_ integrin receptors on cancer cells. Compared to protein- and small thiolate ligand-capped AuNCs, peptide-stabilized AuNCs can offer the following distinct advantages: (1) the variability of the peptide sequence allows fine-tuning of the AuNCs’ particle size, QY, and maximum emission wavelength; (2) the feasibility of manipulating the degree of AIEE of the AuNCs by controlling the pH of the solution; and (3) the incorporation of oppositely charged macromolecules with modified targeting ligands can cause the aggregation of the AuNCs, forming high-brightness and high-selectivity AIEE dots. Yet, further research is needed to understand the correlation between peptide sequence and emission wavelength, aiding the synthesis of NIR II-emitting AuNCs.

## Experimental Section

### Materials and Characterization

All designed peptides were purchased from Synpeptide, Ltd. (Nanjing, China). Detailed information on other chemical substances is provided in the Supporting Information. Additionally, full details of the instruments and conditions used to measure and characterize AuNC are given in the Supporting Information.

### Synthesis of AIEE-Active AuNCs and AIEE Dots

A mixed solution of HAuCl_4_ (20 mM, 500 μL), peptides (20 mM, 500 μL; including ECE, EECEE, EEECEEE, GGCGG, and RRCRR peptides), and TCEP (20 mM, 500 μL) was incubated at 70 °C for 15 min. NaBH_4_ (13.8 mM, 58 μL) was added, and the reaction proceeded at 70 °C for 8 h. The as-prepared peptide-stabilized AuNCs were purified by dialysis with deionized water. The gold concentrations of ECE-, EECEE-, EEECEEE-, GGCGG-, and RRCRR-stabilized AuNCs were 1764, 657, 1258, 1311, and 520 μg/mL, respectively. For synthesizing high-brightness AIEE dots, the EECEE-stabilized AuNCs (50 μL, 65 μg/mL) were incubated with RRRRR (R5) or RGDR5 peptides (50 μL, 0–50 μM) at pH 7.0 in a 10 mM PBS. After they were shaken at room temperature for 10 min, their luminescence properties were measured under 480 nm excitation light.

### pH Sensing in Solutions and Live Cells

A 10-fold dilution of the EECEE-stabilized AuNCs (50 μL) was added to 150 μL of 40 mM PBS (pH 3.0–10.0; 0.2 pH interval), and the mixture was shaken at 37 °C for 10 min. Luminescence spectra of the EECEE-stabilized AuNCs under pH changes were examined by excitation at 480 nm. Experiments concerning cell culture steps and MTT analysis of material toxicity are included in the Supporting Information. After harvesting at 37 °C for 25–30 h, 20–40 μL of HeLa cell solution was transferred into a 35 mm confocal dish. Two mL of culture medium was then pipetted into the confocal dish and incubated in a sterile CO_2_ incubator (37 °C, 5% CO_2_) for 24 h. Subsequently, 33 μg/mL of 200 μL of EECEE-stabilized AuNCs was added to the cell-containing confocal dish, followed by incubation in a sterile CO_2_ incubator for 6 h. After suctioning the culture solution, cells were incubated with 2 mL of a pH calibration curve buffer or 1x PBS buffer (pH 5.0–7.5; 0.5 pH unit interval). Next, cells were washed twice with 2 mL of a pH calibration curve buffer or 1× PBS buffer and then fixed with 2 mL of 4% paraformaldehyde solution for 20 min. Luminescence images of AuNC-labeled cells were captured using CLSM with a 63× oil immersion objective and a 488 nm laser. Two-photon excitation microscopy, employing a Coherent Chameleon Vision II femtosecond laser (680–1600 nm), was used for two-photon luminescence imaging with a laser wavelength set to 840 nm. The luminescence intensity of the AuNCs in live cells was transformed into continuous LUT colors with the help of ImageJ.

### Lysosomal Colocalization Studies

Lysosomal colocalization studies were performed by adding 33 μg/mL of 200 μL of the EECEE-stabilized AuNCs to a cell-containing confocal dish, followed by incubation in a sterile CO_2_ incubator for 0.5–24 h. After the culture medium was discarded, the cells were washed using 1× PBS. The cells were then incubated in confocal dishes for 30 min with 1 mL of culture medium containing 75 nM Lysotracker Green. The cells were washed twice with 2 mL of 1× PBS buffer solution, then fixed with 4% of 2 mL of paraformaldehyde solution for 20 min, and finally washed twice with 2 mL of 1× PBS buffer solution. The fluorescence images of the Lysotracker Green-labeled lysosomes were recorded by a CLSM equipped with a 561 nm laser based on the emission collected at 580–630 nm. At an excitation wavelength of 488 nm, the AuNC-labeled cells were imaged in the 700–800 nm emission region.

### Luminescence Imaging of α_v_β_3_ Integrin Receptor-Positive Cells

The cyclic RGD-modified and R5-containing AIEE dots were incubated with HeLa and MCF-7 cells in culture media (2 mL) in an incubator (5% CO_2_, 37 °C) for 2 h. The obtained cells were washed twice with 2 mL of 1× PBS, fixed with 2 mL of 4% paraformaldehyde for 20 min, and then washed twice with 2 mL of 1× PBS. The labeled cells were imaged using CLSM and two-photon excitation microscopy.

## Supporting Information Available

Additional experimental details associated with Chemicals and buffers, Determination of peptide’s pI values, Characterization of the AuNCs, Luminescence imaging of single AuNCs, cellculture and MTT assay, Tables S1–S3 and Figures S1–S13 (MP4)
(MP4)
(MP4)
(MP4)
(MP4)
(MP4)
(PDF)
(MP4)
