# Supplementary material for: Peptide-Directed Synthesis of Aggregation-Induced Emission Enhancement-Active Gold Nanoclusters for Single- and Two-Photon Imaging of Lysosome and Expressed αvβ3 Integrin Receptors
Source: Anal Chem. 2024 May 23;96(22):9007–15. doi: 10.1021/acs.analchem.4c00321 (PMC11154667; doi:10.1021/acs.analchem.4c00321)
Supplement: Supplementary file 7 — ac4c00321_si_007.pdf [file ac4c00321_si_007.pdf]

Supporting Information

Peptide-Directed Synthesis of Aggregation-Induced Emission Enhancement-Active Gold Nanoclusters for Single and Two-Photon Imaging of Lysosome and Expressed $\alpha_v\beta_3$ Integrin Receptors

Manivannan Madhu,[1] Wei-Bin Tseng,[1,2] Yi-Shiuan Chou,[1] A. Santhana Krishna Kumar,[1,4] Chi-Yu Lu,[3] Po-Ling Chang,[1] Wei-Lung Tseng*[1,5]

1. Department of Chemistry, National Sun Yat-sen University, No. 70 Lienhai Rd., Kaohsiung 80424, Taiwan.

2. Department of Environmental Engineering, Da-Yeh University. No.168, University Road, Dacun, Changhua 515006, Taiwan

3. School of Pharmacy, Kaohsiung Medical University, No. 100, Shiquan 1st Road, Sanmin District, Kaohsiung 80708, Taiwan

4. Faculty of Geology, Geophysics and Environmental Protection, AGH University of Science and Technology, Al. Mickiewicza 30, 30-059, Krakow city, Poland.

5. School of Pharmacy, College of Pharmacy, Kaohsiung Medical University, No.100, Shiquan 1st Rd., 80708, Kaohsiung, Taiwan

Correspondence: Dr. Wei-Lung Tseng, E-mail: tsengwl@mail.nsysu.edu.tw

Fax: 011-886-7-5254644

Experimental Section

Chemicals and buffers. 4-(2-hydroxyethyl)-1-piperazineethanesulfonic acid (HEPES), 2-(N-morpholino)ethanesulfonic acid (MES), NaCl, disodium hydrogen phosphate, sodium dihydrogen phosphate, $NaBH_4$, TECP, MTT, indocyanine green, fluorescein isothiocyanate, and RPMI-1640 medium were purchased from Sigma-Aldrich (St. Louis, MO). Hydrogen tetrachloroaurate(III) trihydrate ($HAuCl_4 \cdot 3H_2O$) was bought from Alfa Aesar (Ward Hill, MA). LysoTracker™ Green DND-26 were bought from Thermo Fisher Scientific (MA, USA). Qdot 800 ITK quantum dots with an emission wavelength of 800 nm (QD800) were acquired from Invitrogen Corporation, CA. A pH calibration curve buffer contained 125 mM KCl, 25 mM NaCl, 10 μM monensin, and 25 mM HEPES (pH 7.5 and 7.0). For pH 6.5–5.0, 25 mM MES was substituted for 25 mM HEPES. Milli-Q ultrapure water (Millipore, Hamburg, Germany) was employed throughout all experiments.

Detemination of peptide's pI values. Compute pI/Mw, available on the ExPASy server (http://www.expasy.org/tools/), was used to predict the pI values of the designed peptides based on their amino acid sequence.

Characterization of the AuNCs. At 200 kV accelerating voltage, the JEM-2100 HRTEM (JEOL, Tokyo, Japan) was used to visualize AuNCs and determine their particle size. The surface state of AuNCs was analyzed using the PHI Quantera SXM XPS system (ULVAC-PHI, Japan). The optical properties of AuNCs were measured with a V670 dual-beam UV-Vis spectrophotometer (JASCO, Tokyo, Japan) and an FS5 fluorometer (Edinburgh Instruments, Livingston, UK). The absolute QY of AuNCs was assessed by integrating an SC-30 integrated sphere into an FS5 fluorometer. The absolute QY of BSA-stabilized AuNCs (~6%) was used to verify the accuracy of the above instruments. The luminescence lifetime of the AuNCs was studied by a time-dependent single-photon counting device (Time-Harp 200, PicoQuant GmbH, Berlin, Germany) in integration with a 390-nm pulsed diode laser (pulse width of tens of seconds). The Nicolet iS5 system (Thermo Fisher Scientific Waltham, MA, U.S.A.) and the N5 submicron particle size analyzer (Beckman Coulter, Inc., U.S.A.) provided information on the functional groups and hydrodynamic diameters of the AuNCs.

The MALDI-TOF-MS (Microflex LT, Bruker Daltonics, Bremen, Germany) spectra of the AuNCs were performed by ionizing the sample with a pulsed

nitrogen laser at 337 nm; 1 μL of 10 mg/mL CHCA was used as a matrix to mix with 1 μL of 657 μg/mL AuNCs. The in-house size exclusion chromatography consists of a UV detector (Thermo, model Spectra system UV2000; detection wavelength of 220 nm), an LC pump (Thermo, model Spectra SERIES P100), a column (OHpak SB-804 HQ), and a mobile phase (30 mM NaCl, 50 mM phosphate buffer, pH 7). A linear calibration curve for quantifying gold ion concentrations was established using an atomic absorption spectrometer (PerkinElmer Analyst 200, USA) equipped with a gold hollow cathode lamp. The calibration curve was applied to determine the gold ion concentration in 100-fold diluted solutions of peptide-stabilized AuNCs. The formation mechanism of the EECEE-stabilized AuNCs was studied by UV-vis absorption spectroscopy, luminescence spectroscopy, Raman spectroscopy (alpha 300R, WITec GmbH, Ulm, Germany; 523 nm laser), and cyclic voltammetry (CHI 760C, CH Instruments, Inc., Austin, TX).

A CLSM (LSM 700, Carl Zeiss GmbH, Jena, Germany) equipped with a 63 oil immersion objective and a 488-nm laser was employed to capture luminescence images of the AuNC-labelled cells in the emission range of 700–800 nm. Two-photon excitation microscopy consisted of a Coherent Chameleon Vision II femtosecond laser (680-1600 nm), an optical parametric oscillator, a ZEISS AXIO IMAGER A2M upright microscope, a 40× objective (N.A. 1.0), and a 3D MEM image system V2.1. Two-photon luminescence images of the AuNC-labelled cells were recorded as the laser wavelength was set to 840 nm.

Luminescence imaging of single AuNCs. The slides were first shocked in deionized water for 5 min, then ultrasonically shocked in 1 M HCl for 30 min, and then ultrasonically shaken in deionized water for 5 min. The resultant slides were placed in 1 M NaOH, followed by repeating the above steps to complete the cleaning of the slides. The cleaned slides were soaked in a mixture (1 mL of 99% APTES and 199 mL of 95% ethanol) for 1 h. Next, the slides were ultrasonically shocked with ethanol for 5 min twice and then dried in an oven. 637 μg/mL EECEE-stabilized AuNCs were diluted 10,000 times with different pH solutions (pH 3.0-9.0), then spotting the diluted solutions on the slides. An inverted microscope (Olympus, Tokyo, Japan) equipped with a UPLanFL Ph3, 100x, NA=1.10 objective, a 488 nm laser, and an EM-CCD camera (ProEM: 512B,

Princeton Instruments) was used to image single particle luminescence of the EECEE-stabilized AuNCs. The filter set included a dichroic mirror and an emission bandpass filter (700–800 nm). In addition, we employed the ImageJ program to analyze the luminescence intensity histograms of individual molecules (National Institutes of Health, USA).

Cell culture. The cervical cancer HeLa cell line was obtained from the Food Industry Research and Development Institute in Hsinchu, Taiwan. Primary cells (30 μL) were cultured in Dulbecco's modified Eagle medium (DMEM) (5 mL; Gibco BRL, Grand Island, NY, U.S.A.) and incubated in a humidified incubator at 37 °C with 5% CO_2. HeLa cells were cultured in T-25 flasks until reaching approximately 80% confluence, which typically took 5–7 days. Upon reaching the desired confluence, adherent cells were washed twice with 1× phosphate-buffered saline (PBS, 4 mL each time), followed by incubation with trypsin-EDTA solution (0.25% w/v trypsin and 0.25 g/L EDTA) at 37 °C for 10 min. The detached cells were collected by centrifugation at 3000 rpm for 5 min, rinsed with DMEM (2 mL), and re-suspended in DMEM (5 mL). Subsequently, the HeLa cells were cultured for an additional 24 h under the same conditions.

Similarly, the cervical cancer MCF-7 cell line was obtained from the Food Industry Research and Development Institute in Hsinchu, Taiwan. Primary cells (30 μL) were cultured in RPMI 1640 Medium (500 mL; Thermo Fisher Scientific Inc., U.S.A.) and incubated in a humidified incubator at 37 °C with 5% CO_2. MCF-7 cells were cultured in T-25 flasks until reaching approximately 80% confluence, which typically took 5–7 days. Upon reaching the desired confluence, adherent cells were washed twice with 1× PBS (4 mL each time), followed by incubation with trypsin-EDTA solution (0.25% w/v trypsin and 0.25 g/L EDTA) at 37°C for 10 min. The detached cells were collected by centrifugation at 3000 rpm for 5 min, rinsed with RPMI 1640 Medium (2 mL), and re-suspended in RPMI 1640 Medium (5 mL). Subsequently, the MCF-7 cells were cultured for an additional 24 h under the same conditions.

MTT assay. HeLa cells (20 μL) were seeded into a 24-well culture plate and incubated with RPMI 1640 medium for 24 h at 37 °C. The EECEE-stabilized AuNCs and AIEE dots at various concentrations were added to each well and incubated at 37 °C for 24 h. After washing away excess aggregates, MTT solution (20 μL, 5

mg/mL) was added to each well and incubated for 4 h at 37 °C. Following further washes, formazan crystals were dissolved with DMSO (300 µL, 98%) and culture medium (125 µL). Absorbance was measured at 570 nm using a microplate reader (BioTek ELX800, BioTek Instruments Inc., Vermont, USA).

Table S1. Optic properties of previously reported AIEE-active MNCs

NCs	Capping agent	λ_{ex}[a] (nm)	λ_{em}[a] (nm)	QY (%)	Ref.
Au	Glutathione	400	627	1.2	Anal. Chim. Acta 2019, 1078, 101-111.
Au	Isoleucine, MUA	295	620	4.2	Anal. Chim. Acta 2019, 1079, 192-199.
Au	Glutathione	360	570	-	Anal. Chim. Acta 2019, 1046 ,170-178
Au	Glutathione	~380	564	1.2	Biosens. Bioelectron, 2021, 193,113522
Au	Glutathione	275	650	4.86	Microchem. J. 2022, 183, 108049
Au	Glutathione	380	564	-	Spectrochim. Acta A Mol. Biomol. Spectrosc. 2024, 304, 123255
Ag	Glutathione	~380	~640	12	Anal. Chem. 2017, 89, 4994–5002
Cu	Glutathione	~380	~596	1.96	Analyst, 2018, 143, 3068–3074
Cu	Glutathione	375	617	0.3	ACS Sustainable Chem. Eng. 2023,11, 1995–2004
Cu	Glutathione	340	612	0.45	Small 2013, 22, 3873–3879
Cu	NH_2NH_2 , 4-methylthiophenol	330	605	3.8	Analyst, 2020, 145, 7009–7017
Au/Ag	Adenosine monophosphate	340	550	8.46	Microchim. Acta, 2020, 187:41

[a]MUA, 11-mercaptoundecanoic acid; AMP, adenosine 5′-monophosphate,

Table S2. Chemical structures and isoelectric point (pI) of the selected peptides for synthesizing AuNCs.

Peptide	pI	Structure
ECE	3.80	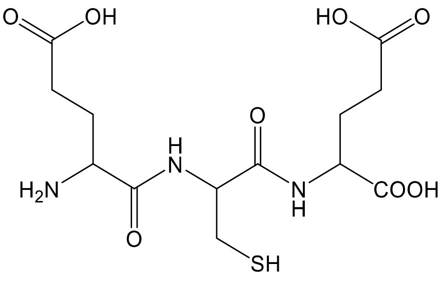
EECEE	3.58	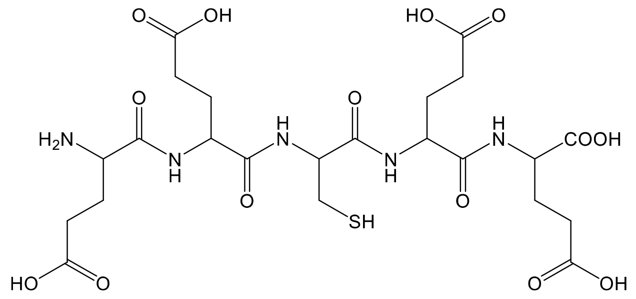
EEECEEE	3.46	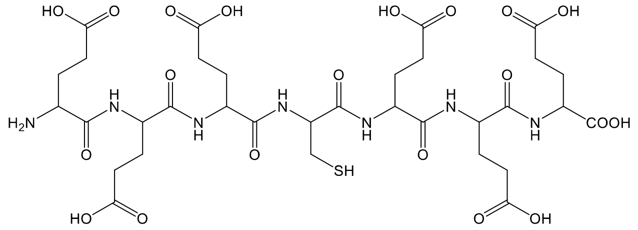
GGCGG	5.52	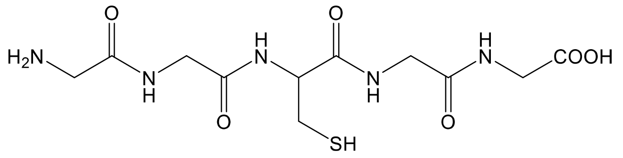
RRCRR	12.0	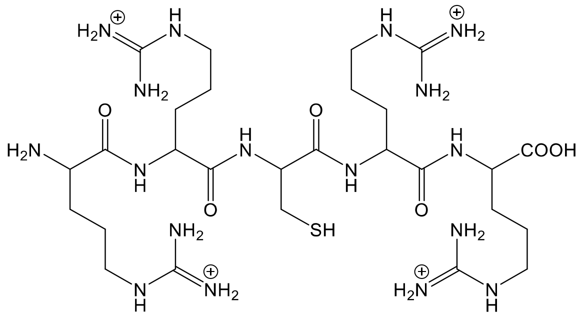

Table S3. Comparison of peptide-stabilized AuNCs and AIEE dots in terms of optical properties and morphology

**Peptide**	**λ_{ex}[a] (nm)**	**λ_{em}[a] (nm)**	**Particle size (nm)**	**D_h[a] (nm)**	**QY (%)**	**Average lifetime (μs)**
ECE-stabilized AuNCs	420	770	1.7 ± 0.3	2.1 ± 0.5	7.0	1.3
EECEE-stabilized AuNCs	380	745	1.0 ± 0.2	1.1 ± 0.2	14	1.2
EEECEEE-stabilized AuNCs	380	740	1.2 ± 0.3	1.5 ± 0.2	7.9	0.45
GGCGG-stabilized AuNCs	400	775	1.1 ± 0.2	1.3 ± 0.3	5.6	0.73
RRCRR-stabilized AuNCs	340	760	2.7 ± 0.7	3.2 ± 1.4	1.8	0.65
R5-containing AIEE dots	380	734	170.5 ± 61.2	215.3 ± 59.5	24	2.1
Cyclic RGD-modified AIEE dots	380	735	184.2 ± 72.5	256.5 ± 73.8	23	2.0

[a] λ_{ex}, maximum excitation wavelength; λ_{em} maximum emission wavelength; D_h, hydrodynamic diameter.

Table S4. Comparison of the EECEE-stabilized AuNCs with the other previously reported pH-sensitive AuNCs.

**Probe/Maximum emission wavelength of AuNCs**	$\boldsymbol{\lambda_{em}}$**[a] (nm)**	**Linear range**	**pH resolution**	**Reference**
Chitosan-modified AuNC aggregates	~610	7.5 to 6.5	0.1	Anal. Chem 2019, 91 (13), 8237-8243.
Hydroxypropyl-β-cyclodextrin-derived AuNC aggregates	625	5.0 to 6.2	0.1	ACS Mater. Lett. 2022, 4 (11), 2244-2251.
BSA-stabilized Ce/Au nanoclusters	650	6.0 to 9.0	0.5	Chem. Commun. 2014, 50 (62), 8571-8574.
AuNC-loaded lysozyme nanoparticles	676	7.5 to 9.5	0.2	J. Mater. Chem. B 2019, 7 (24), 3876-3883.
Fluorescein isothiocyanate-conjugated BSA-stabilized AuNCs	670	6.0 to 8.0	0.5	Nanoscale 2016, 8 (21), 11210-11216
EECEE-stabilized AuNCs	745	5.0 to 8.0	0.2	This study

[a] λ_{em} maximum emission wavelength of AuNCs

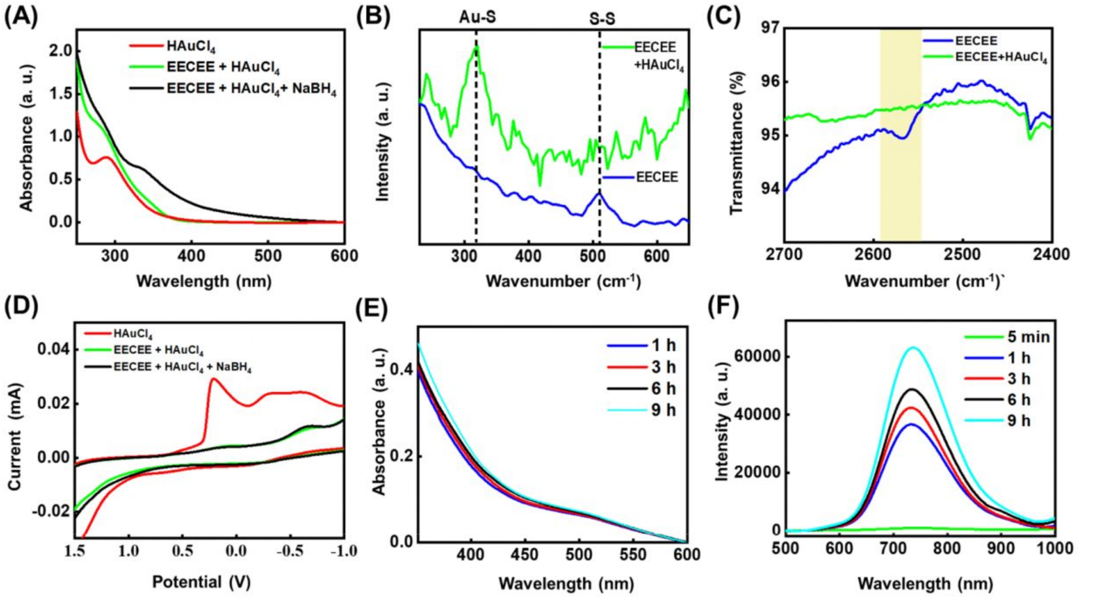

Figure S1. Spectroscopic characterization of the formation of the EECEE-stabilized AuNCs. (A) UV-Vis absorption spectra, (B) Raman spectra, (C) FT-IR spectra, and (D) CV voltammograms of (A, D) $HAuCl_4$ (red line), (B, C) TCEP-treated EECEE (blue line), (A–D) TCEP-treated EECEE peptide with $HAuCl_4$ (green line), and (A, D) $NaBH_4$-mediated reaction of TCEP-treated EECEE and $HAuCl_4$ for 5 min (black line). (E, F) Time-evolution (E) absorption and (F) fluorescence spectra of the products obtained from the $NaBH_4$-mediated reaction of TCEP-treated EECEE and $HAuCl_4$ for (E) 1, 3, 6, and 9 h and (F) 5 min, 1 h, 3 h, 6 h, and 9 h. The Raman peaks of the disulfide and Au-S bond were reported to be located at 510 cm^{-1} (referred to PloS one 2012, 7, e36989) and 320 cm^{-1} (referred to Nanoscale 2012, 4, 531-540), respectively.

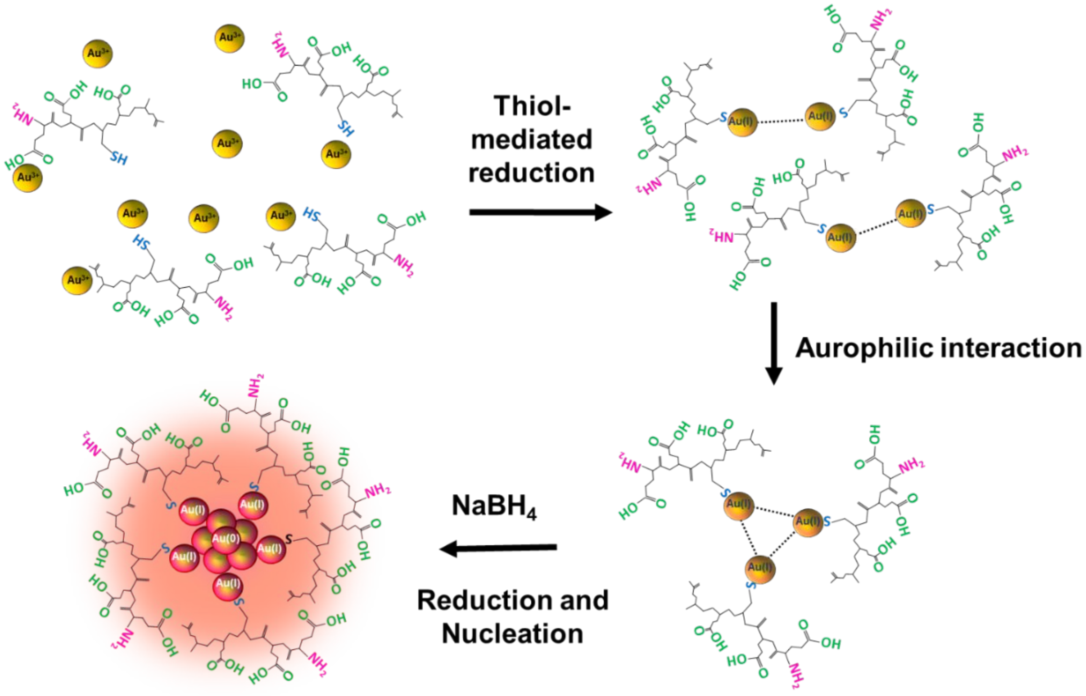

Figure S2. Schematic illustration of the stepwise process involved in creating the EECEE-stabilized AuNCs, starting from reduction and nucleation to particle growth.

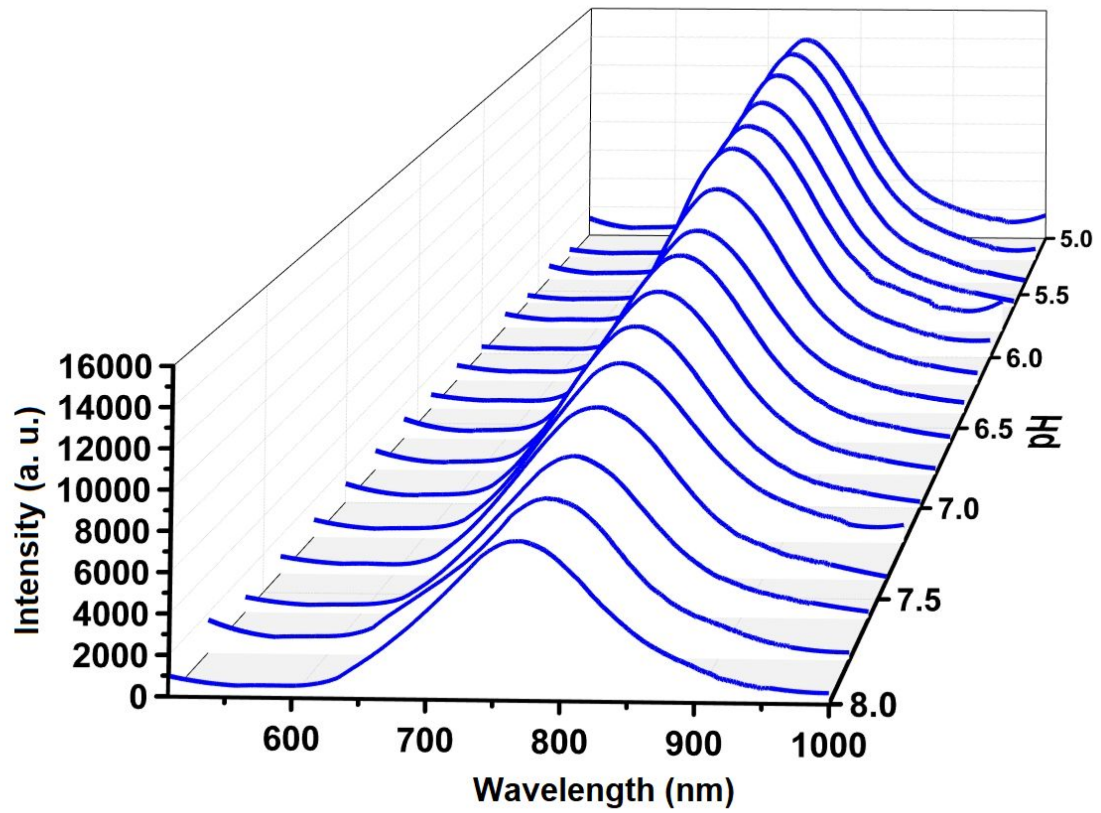

Figure S3. Effect of solution pH on the luminescent spectra of the EECEE-stabilized AuNCs.

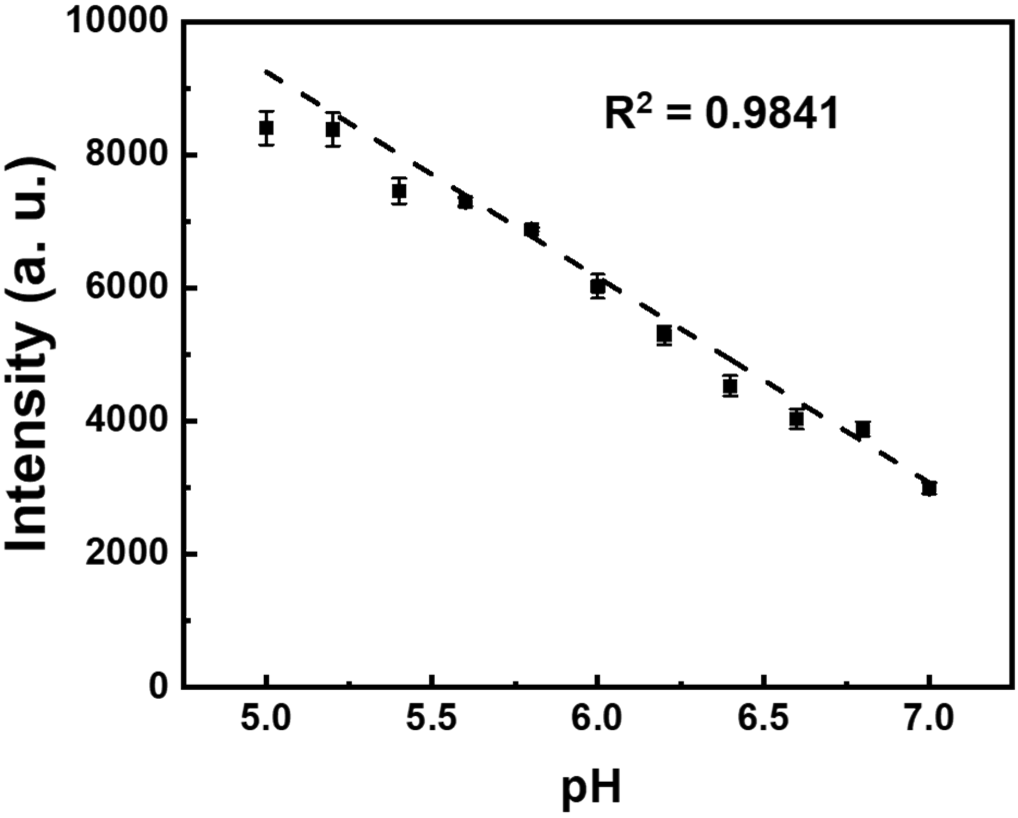

Figure S4. A plot of the pH of a solution versus the fluorescence intensity of the EECEE-stabilized AuNCs at 745 nm in the presence of 5 mM glutathione and 5 mM adenosine triphosphate.

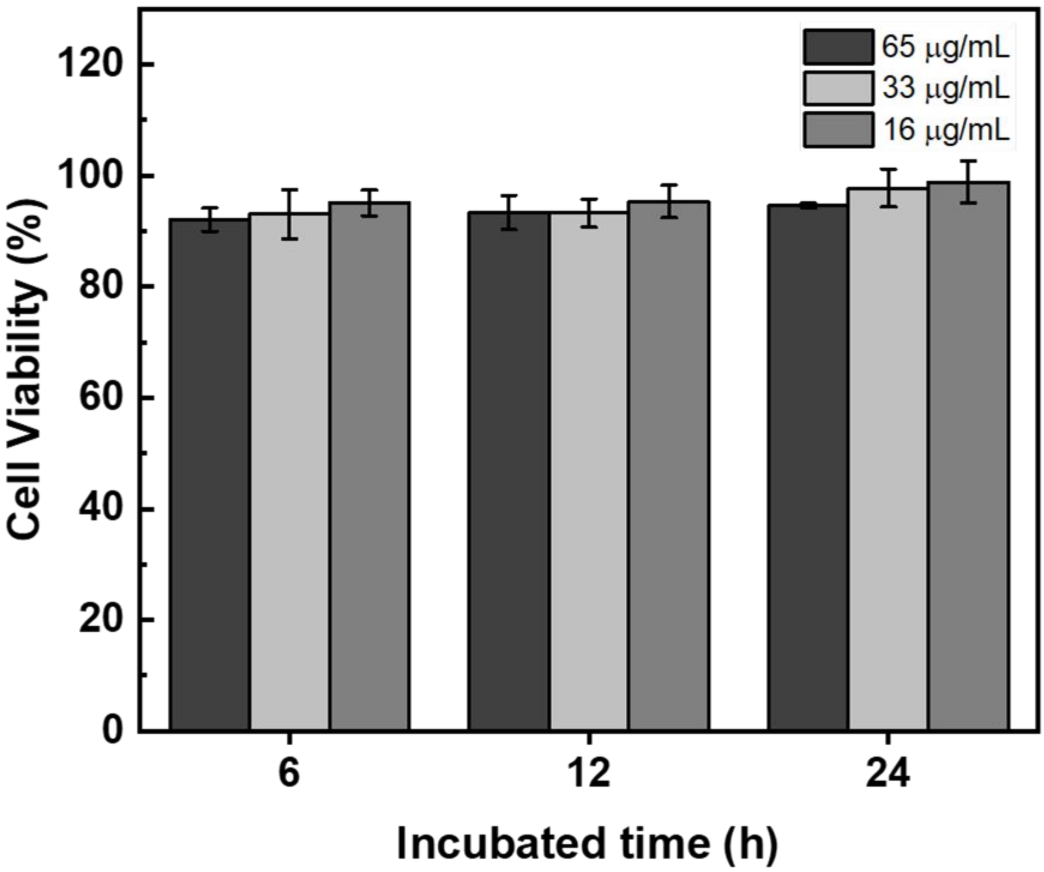

Figure S5. The viability of HeLa cells after incubation with 65 μg/mL, 33 μg/mL, and 16 μg/mL EECEE-stabilized AuNCs.

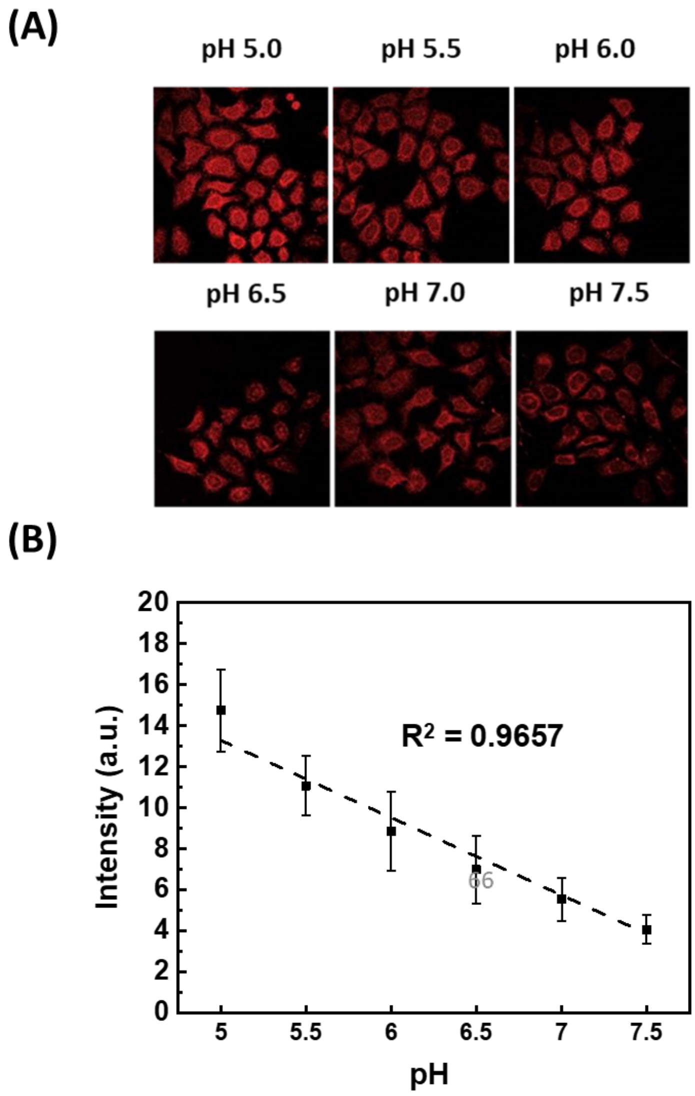

Figure S6. Luminescence imaging of intracellular pH changes with the EECEE-stabilized AuNCs. (A) CLSM images of HeLa cells cultured with 1x PBS buffer at different pH, followed by labeling with the EECEE-stabilized AuNCs. (B) Mean intracellular luminescence intensity obtained from the CLSM images of the labeled HeLa cells (n = 100) at different pH values. The excitation wavelength was set to 488 nm. The luminescence of the EECEE-stabilized AuNCs was collected in the NIR channel (700-800 nm).

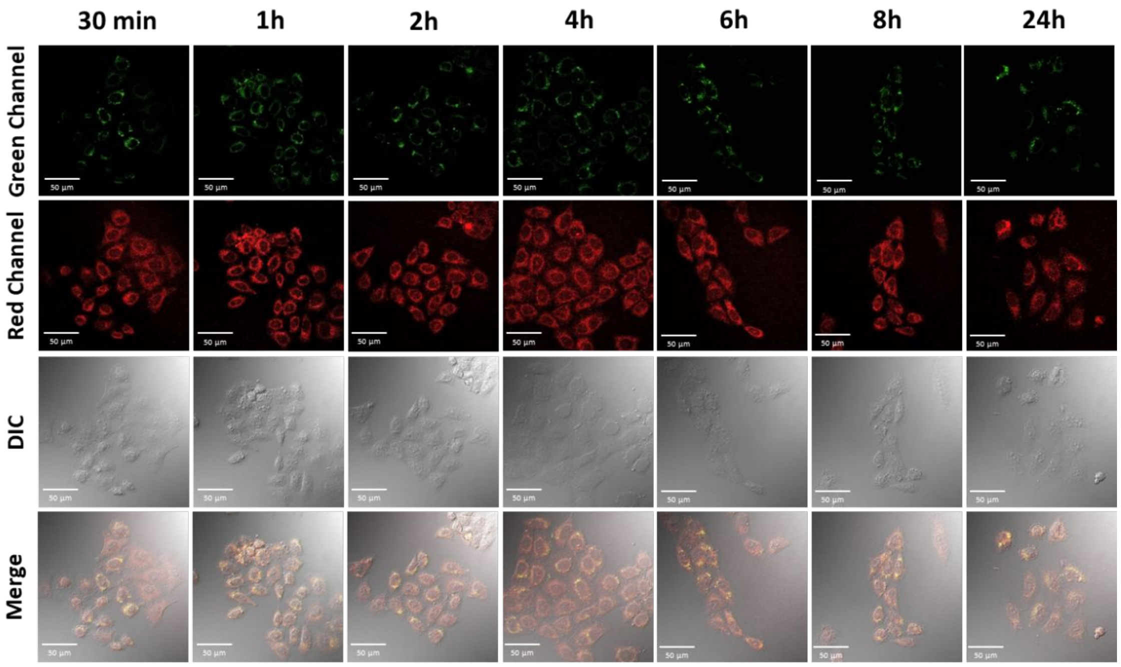

Figure S7. Colocalization of lysosomes in HeLa cells labeled with the EECEE-stabilized AuNCs and Lysotracker Green. CLSM images of the labeled HeLa cells collected from green and red channels. The merged image obtained by overlapping the green channel CLSM image, red channel image, and DIC image of labeled HeLa cells.(Scale bar 50 μM)

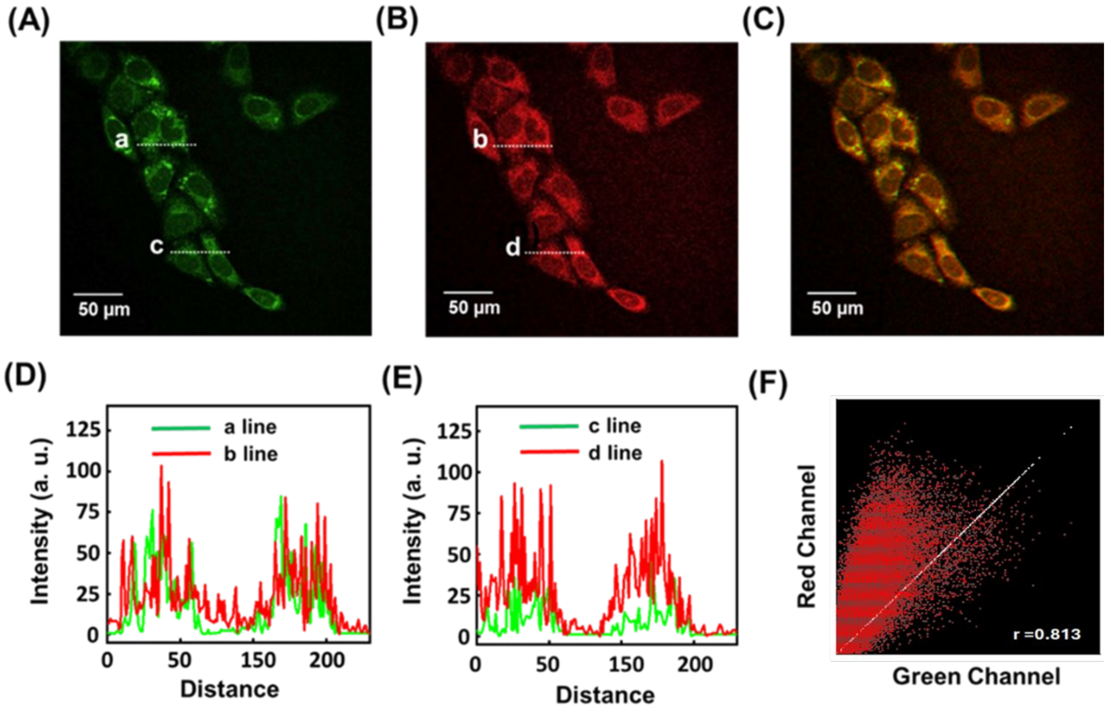

Figure S8. Colocalization of lysosomes in HeLa cells labeled with the EECEE-stabilized AuNCs and Lysotracker Green. (A, B) CLSM images of the labeled HeLa cells collected from (A) green and (B) red channels. HeLa cells were treated with the EECEE-stabilized AuNCs for 6 h, followed by Lysotracker Green staining. (C) The merged image obtained by overlapping the green-channel and red-channel CLSM images of labeled HeLa cells. (D) Intensity profiles along straight white lines a in (A) and b in (B). (E) Intensity profiles along straight white lines c in (A) and d in (B). (F) The intensity scatter plot of the two channels. The excitation wavelength was set to 488 nm. The luminescence of the EECEE-stabilized AuNCs and fluorescence of Lysotracker Green were collected in the green (500-600 nm) and NIR channels (700-800 nm), respectively.

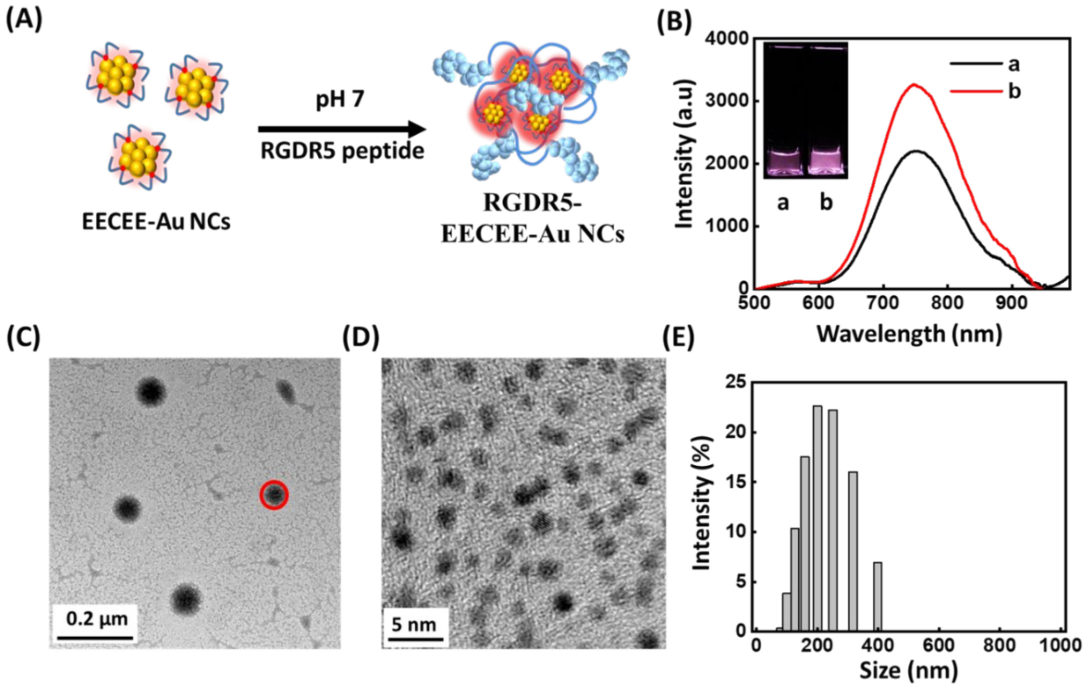

Figure S9. The Formation of the cyclic RGD-modified AIEE dots. (A) Schematic illustration associated with the formation of the cyclic RGD-modified AIEE dots by mixing cyclic RGDR5 with the EECEE-stabilized AuNCs. (B) Luminescence spectra and photographs of the EECEE-stabilized AuNCs (a) before and (b) after the addition of cyclic RGD peptides. The EECEE-stabilized AuNCs (16 μg/mL) were incubated with 35 μM cyclic RGDR5 in 10 mM phosphate buffer (pH 7.0) for 10 min. The excitation wavelength was set to 488 nm. (C) TEM image, (D) enlarged TEM image (the red circle in C), and (E) DLS measurement of the cyclic RGD-modified AIEE dots.

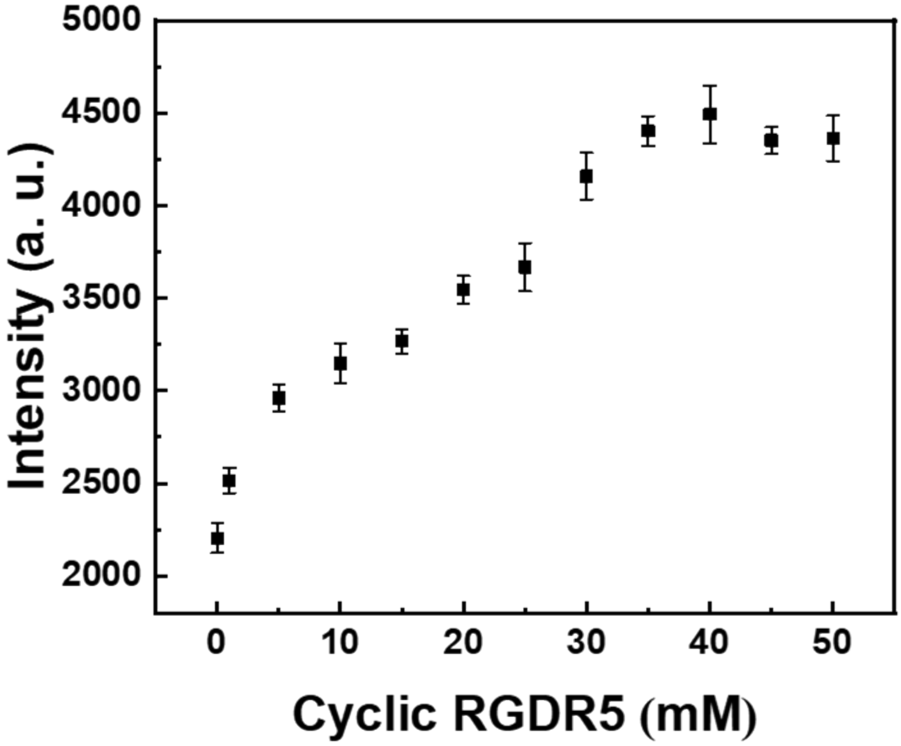

Figure S10. Effect of the cyclic RGDR5 concentration on the luminescence intensity of the EECEE-stabilized AuNCs. The EECEE-stabilized AuNCs (16 μg/mL) were incubated with different concentrations of cyclic RGDR5 in 10 mM phosphate buffer (pH 7.0) for 10 min. The excitation wavelength was set to 488 nm.

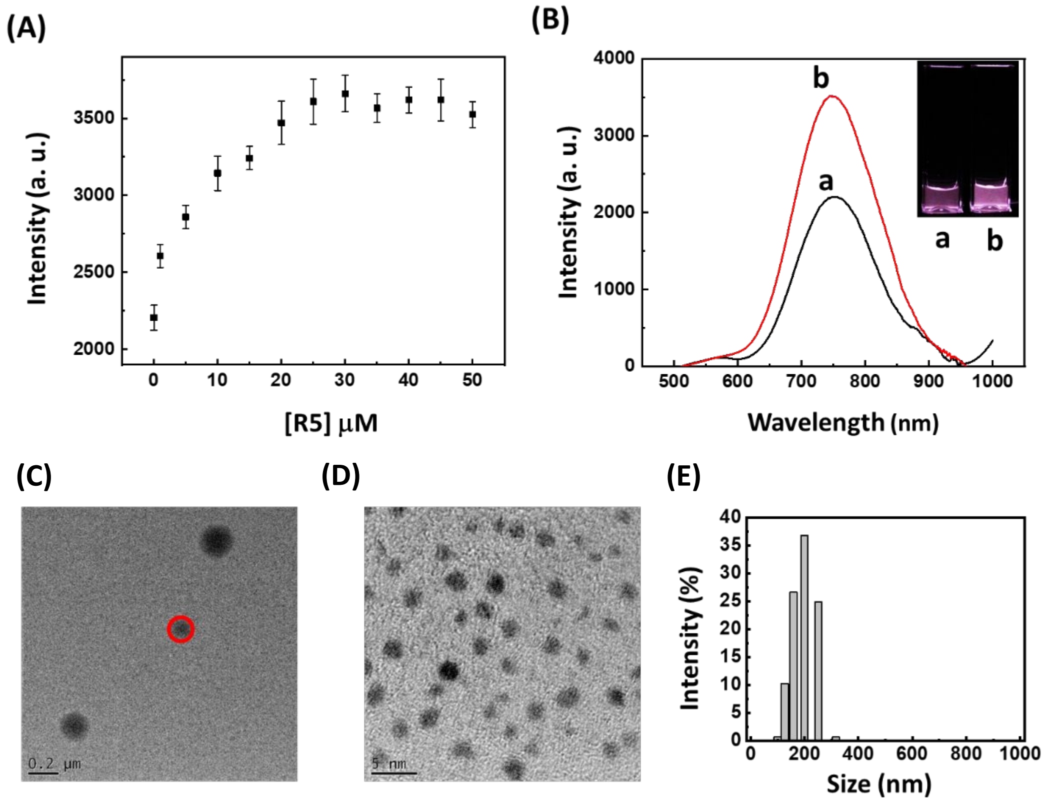

Figure S11. The Formation of the R5-containing AIEE dots. (A) Effect of the R5 concentration on the luminescence intensity of the EECEE-stabilized AuNCs. (B) Luminescence spectra and photographs of the EECEE-stabilized AuNCs (a) before and (b) after adding R5 peptides. (A, B) The EECEE-stabilized AuNCs (16 μg/mL) were incubated with (A) 0–50 μM and (B) 35 μM R5 in 10 mM phosphate buffer (pH 7.0) for 10 min. The excitation wavelength was set to 488 nm. (C) TEM image, (D) enlarged TEM image (the red circle in C), and (E) DLS measurement of the R5-containing AIEE dots.

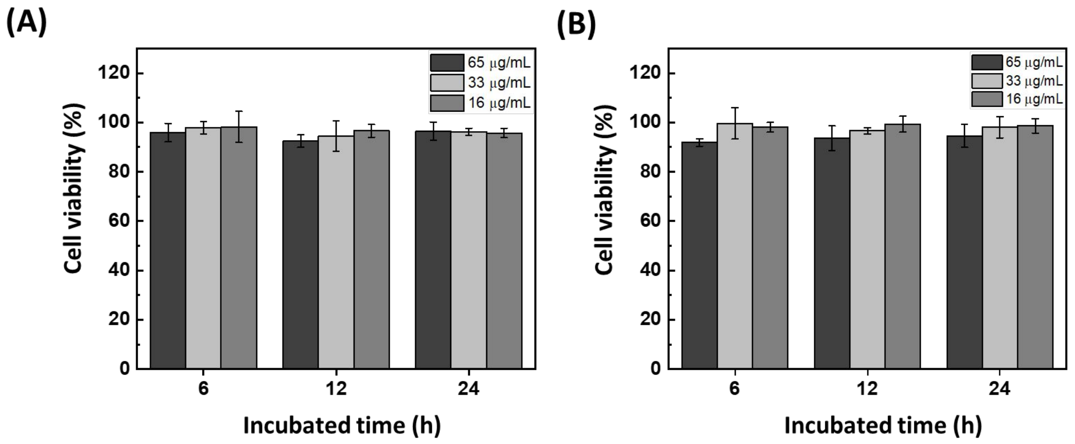

Figure S12. The viability of HeLa cells after incubation with 65 µg/mL, 33 µg/mL, and 16 µg/mL of (A) the R5 containing AIEE dots and (B) the cyclic RGD-modified AIEE dots.

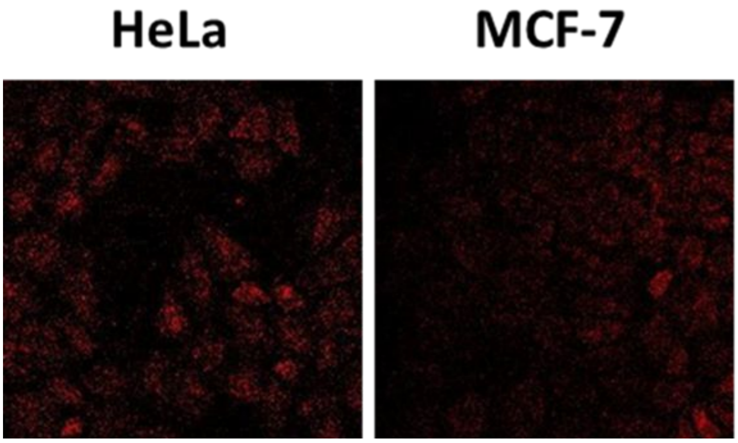

Figure S13. Two-photon images of HeLa and MCF-7 cells labeled with cyclic RGD-modified AIEE dots. The excitation wavelength was set to 880 nm. The luminescence of the EECEE-stabilized AuNCs was collected in the NIR channel (700-800 nm).
